# X-Phi and Carnapian Explication

**DOI:** 10.1007/s10670-014-9648-3

**Published:** 2015-04-01

**Authors:** Joshua Shepherd, James Justus

**Affiliations:** Oxford Centre for Neuroethics, University of Oxford, 16-17 St. Ebbes Street, Oxford OX1 1PT, UK; Department of Philosophy, Florida State University, 151 Dodd Hall, Tallahassee, FL 32306-1500, USA

## Abstract

The rise of experimental philosophy (x-phi) has placed metaphilosophical questions, particularly those concerning concepts, at the center of philosophical attention. X-phi offers empirically rigorous methods for *identifying* conceptual content, but what exactly it contributes towards *evaluating* conceptual content remains unclear. We show how x-phi complements Rudolf Carnap’s underappreciated methodology for concept determination, explication. This clarifies and extends x-phi’s positive philosophical import, and also exhibits explication’s broad appeal. But there is a potential problem: Carnap’s account of explication was limited to empirical and logical concepts, but many concepts of interest to philosophers (experimental and otherwise) are essentially normative. With formal epistemology as a case study, we show how x-phi assisted explication can apply to normative domains.

## 1 Introduction

Identifying and evaluating conceptual content is at the core of philosophical practice. Concepts are indispensable constituents of arguments and theories, and conceptual judgments often motivate or undermine the development of both. Moreover, subtle disagreements about conceptual content often underlie much broader philosophical debates. But despite its fundamental role, the rules of engagement for disputes over conceptual content remain obscure.^1^

There is a received view. Traditional conceptual analysis places principal weight on intuitive judgments across possible scenarios. Such judgments are taken to establish parameters that successful accounts of concepts must respect. Intuitions regarding a concept C thereby function as primary data for which analyses of C account.^2^

Adherence to this approach has been unsettled by serious doubts about the epistemic import of intuition, doubts exacerbated by data-driven approaches to conceptual issues championed by experimental philosophers (see Weinberg et al. 2001; Weinberg 2007). But these concerns, however compelling, leave critical questions about proper *positive* philosophical methodology unaddressed. Principally: if not by intuition, how could (and should) conceptual content be identified and evaluated?

Rudolf Carnap formulated a compelling answer: *explication*. The general philosophical significance of explication has recently been rearticulated and defended (see Carus 2007; Maher 2007; Kitcher 2008; Justus 2012). This paper enhances and extends the case in two ways. First, we identify an unrecognized consilience between explication and experimental philosophy (x-phi), one that clarifies the philosophical import of the latter. Second, we diagnose a deficiency in Carnap’s original account of explication, and show how it can be fixed.

Section 2 begins by discussing the controversy over x-phi’s contribution to philosophy, one which divides experimental philosophers themselves. We criticize the so-called positive program for x-phi as it is commonly characterized, and argue that explication furnishes a valuable new role for x-phi. Section 3 develops a novel positive program for x-phi we call *explication preparation*, which Carnap (1955) himself seems to have anticipated. In brief, x-phi supplies the necessary clarification of conceptual content that constitutes the raw material for explication. In so doing, x-phi facilitates the explicative evaluation of conceptual content.

Section 4 uncovers a deficiency in Carnap’s rationale for explication, one that poses a potential limitation for this methodological marriage. In continuity with scientific practice, Carnap considered explications valuable to the extent they enhanced what he called ‘fruitfulness.’ For empirical concepts, fruitfulness was measured in the same underlying currency in which science measures epistemic success: well-confirmed generalizations. But many concepts of philosophical interest are decidedly normative. Carnap said nothing about how fruitfulness should be conceptualized for such concepts. Using the epistemically normative concepts of formal epistemology as a case study, we illustrate the severity of the challenge. Section 5 shows how x-phi assisted explication can be fruitful for normative domains. As an auspicious consequence, formal epistemology can avoid overreliance on intuition, and thereby the fate of traditional conceptual analysis.

## 2 X-Phi’s Philosophical Value

X-phi privileges empirical data over other sources of information about concepts and selectively appropriates experimental methods used by psychologists to acquire it. The typical protocol involves asking laypeople to apply concepts to specific questions about carefully concocted scenarios (Did X *intentionally* A? Does X *know* that p?). Replacing speculation about the conceptual judgments people *would* make with data about the judgments people *actually do* make is the overriding agenda. The results have yielded surprising, counterintuitive, and controversial information about concepts such as intentional action (Knobe 2003) and knowledge (Weinberg et al. 2001), among others.

Surveys have thereby proved valuable, but they do not exhaust x-phi’s purview. X-phi should be understood in much broader terms; its focus, scope, and methods continue to evolve. For example, more sophisticated statistical measures and methods are becoming commonplace in x-phi as collaboration increases between experimental philosophers and psychologists. In fact, the research programs of many psychologists—which involve methods that go far beyond surveys—address philosophical issues (e.g. Greene 2003 and Malle 2004), and it is unclear what distinguishes this work from x-phi other than disciplinary label. In general, x-phi “takes the concerns with moral and conceptual issues that have so long been associated with philosophy and connects them with the use of systematic and well-controlled empirical investigations that one more typically finds in psychology” (Knobe et al. 2010, 157).

This characterization captures x-phi’s empirical focus and underscores the fact that surveys constitute but one facet of the program. But it also exposes a metaphilosophical lacuna that could threaten x-phi’s philosophical value. How can empirical data, which *describe* concepts, play any direct role in the *normative evaluation and determination* of conceptual content usually thought to be the proper (and primary) purview of philosophy? Critics have exploited this lacuna in various ways.

Kauppinen (2007) distinguishes between *surface* and *robust* intuitions, and argues x-phi only measures the former. Philosophy apparently only concerns the latter: intuitions of competent participants operating in sufficiently ideal conditions where only semantic (as opposed to pragmatic) considerations matter (2007, 97). Since, Kauppinen says, only philosophical dialogue can elicit robust intuitions, philosophical claims “cannot be tested with methods of positivist social science” (2007, 95). Although x-phi results require explanation, Kauppinen maintains it is explanation of a primarily *psychological* rather than *philosophical* kind (2007, 108).

For Jackson (2011), x-phi results are relevant for determining what concepts we *actually* have. But he argues that much of philosophy is concerned with the concepts we *should* have. While x-phi results might constitute “an essential first step in the discussion of the normative question” (481), Jackson holds that “polls won’t be relevant to assessing the final product” (480).

Sosa (2008) argues x-phi results fail to undermine the role of intuition in philosophy, which analogically functions (he suggests) as observation functions in empirical science. Intuitions provide access to the proper subject matter of philosophical inquiry, for example, the “evaluative or normative truths of epistemology” (239). Sosa considers the possibility that “some crucial difference between natural phenomena and evaluative phenomena…rules out any such analogy,” (239) but concludes that even if it did philosophy, not x-phi, would point the way forward. Sosa’s rhetorically asks: “how could we possibly approach such a question except philosophically, through the sort of reflection plus dialectic that depends crucially on philosophical intuition?” (239).

These criticisms exhibit subtle differences but share a common worry. Put bluntly, the worry is that the phenomena motivating psychological theorizing (and x-phi) are distinct *in kind* from what motivates philosophical theorizing. One is a strictly descriptive enterprise, the other is essentially normative. If so, the data x-phi generates and analyzes cannot dictate—perhaps in principle or even partially—the content of philosophical theories.

The worry is acute. Experimental philosophers themselves are concerned with demonstrating the philosophical import of their discipline but divided on how to do so, prompting Nadelhoffer and Nahmias (2007, 129) to call x-phi “a house divided.” Unsurprisingly, the proper role of intuition in identifying and evaluating conceptual content is the main dispute.^3^ Two views have emerged, the so-called *positive* and *negative programs* (Alexander et al. 2010).^4^ As currently conceptualized, the former gives intuition a central role; the latter downplays its significance.

Different reactions to data on the well-known Knobe effect indicate how the programs diverge. In x-phi’s early days, Knobe (2003) discovered a surprising asymmetry regarding conceptions of intentional action. When an agent brings about a negative side effect A of performing an action, most people say A was done intentionally. But when an agent brings about a positive side effect B, most people say B was done unintentionally. This asymmetry is striking and was initially thought to reveal something significant about the folk concept of intentional action. Yet subsequent research shows the asymmetry is subject to order and priming effects (Mele and Cushman 2008), is moderated by individual personality differences (Feltz and Cokely 2009), and that a significant minority of folk manifest no asymmetry (Mele and Cushman 2008). Beyond the folk, these kinds of patterns have also been found among professional philosophers (Schwitzgebel and Cushman 2012).

For negative program proponents, these kinds of results constitute strong evidence that intuitions rarely (if ever) provide trustworthy data about concepts. For intentional action specifically, the conceptual vicissitudes revealed seem to impede rather than advance a positive analysis of the concept. And this is not an isolated case. Far from revealing concepts with unequivocal content, x-phi generally uncovers unprincipled patterns in conceptual judgments. For example, many folk reject the apparently highly intuitive principle that one knows p only if one believes p (Myers-Schulz and Schwitzgebel 2013). Intuitions about scenarios involving concepts of interest to philosophers appear to display the same cognitive diversity studies have revealed in other contexts (Weinberg et al. 2001). Rather than complement the traditional philosophical emphasis on intuition, x-phi seems to be subverting it.

With varying degrees of vigor, positive program proponents reject this implication. Instead, a two-step method is usually defended. For a given concept C, the first task is developing a theory explaining the psychological processes responsible for intuitions about C (once those intuitions have been systematically collected and assessed). Second, the theory is used to vet those intuitions: to inform judgments about their epistemic significance. The idea is that “[i]t is precisely when we are aware of the features our intuitions track that we are able to reflectively criticize whether these intuitions are warranted, and whether these intuitions should carry weight in a mature philosophical account” (Sripada and Konrath 2011, 375).

The first step has obvious value, and is one to which x-phi makes a significant contribution (see Sect. 3). Our complaint concerns the philosophical weight accorded intuition in the second step. Consider, for example, how Beebe and Buckwalter (2010) interpret data that suggest the folk are more likely to claim an agent *knew* a side effect of her action would occur if the effect was negative (e.g. harming the environment) rather than positive (e.g. helping the environment). After discussing five potential explanations of psychological processes responsible for these intuitions, they endorse a model that holds the intuitions “arise from general facts about the relationship between folk psychology and normative assessment rather than from features that are unique to the individual concepts in question” (493). Next, and crucially, this model is taken to support philosophical claims about knowledge, such as: “whether a subject knows that p may depend upon the moral status of actions the subject performs in light of the belief that p” (495). On this approach, proper determination of the ‘knowledge’ concept responds largely to the best psychological theory of folk intuitions about knowledge.

If pinpointing how humans *currently* deploy concepts is the goal, this empirically-driven methodology has obvious promise. But the second step requires a methodology for evaluating conceptual content and determining the concepts we *should* have. And in this regard, the positive program needs development. The problem is the continued emphasis on intuition. Understanding psychological processes responsible for intuitions about concepts may identify potential sources of cognitive error, but how this understanding lends prescriptive force to intuitions that remain even after such errors have been corrected is opaque.

In effect, the shared focus on intuition aligns the positive program with traditional conceptual analysis: “[the positive program] shares with traditional armchair philosophy that intuitions about X are a trustworthy source of evidence or data for philosophical theorizing about X” (Alexander et al. 2010, 300). As such, though x-phi stresses intuitions be gathered in controlled, statistically rigorous, and systematic ways, conceptual analysis and the positive program as it is typically understood share a similar philosophical agenda and fate. On both approaches intuitions regarding hypothetical scenarios are intended to serve their traditional function: justify purported counterexamples to analyses of concepts, support premises, and shift the burden of proof in philosophical debates.^5^ But this alignment exposes the positive program to now well-known criticisms (see Weinberg et al. 2012):

(i) Intuitions are but one source of data about concepts and other sources—e.g., studies of usage drawn from linguistics and anthropology, as well as investigations of the psychological sources of intuition (see Scholl 2007)—are likely to provide more reliable information. Absent a compelling argument that intuitive considerations (even suitably empirically vetted) somehow penetrate to the heart of things while empirically scrutable data sources from anthropology, linguistics, sociology, and elsewhere fall short, it is far from clear intuitions deserve the privileged cognitive status they are often accorded. How they alone, primarily, or significantly lend prescriptive force to philosophical theories is particularly unclear.
(ii) Intuition-based traditional conceptual analysis has a shoddy epistemic track record (Harman 1994). That intuition often leads *empirical* inquiry astray is unsurprising (Ladyman and Ross 2007). But history also indicates intuition unreliably guides *conceptual* inquiry. As a prominent proponent of conceptual analysis recently noted, “The problem for armchair philosophy…is the actual or potential disagreement that pervades our field. This is not disagreement that pits experienced philosophers against street-corner respondents. It is rather the longstanding, well-known disagreement among the ‘experts’ themselves” (Sosa 2011, 462). Intuitions are supposed to function as evidence for or against analyses of concepts. Yet intuitions chronically conflict. Short of a reliable, non-intuition-based method for adjudicating such conflict, the status of intuitions as evidence for philosophical theories is therefore suspect.
(iii) Different populations perceive and reason about the world in significantly different ways (see Heinrich et al. 2010). This is unproblematic if conceptual commonalities obtain despite such cultural differences, or if such differences do not influence intuitions regarding concepts of interest to philosophers. But the evidence seems to tell against both possibilities: it looks like cultural differences often lead to systematically different and incompatible intuitions (see Zarpentine et al. 2012). This undermines conceptual analysis as a prescriptive philosophical methodology, particularly its aspiration to deliver universal conceptual truths.

Much research in the positive program is motivated by the assumption—often drawn from assertions of philosophers outside x-phi—that “what the folk say” is pivotally important to philosophical debates. As Nadelhoffer and Nahmias note, these experimental philosophers “essentially agree with many traditional philosophers…about the *relevance* of folk intuitions; they simply disagree about the best *methods* for getting at these intuitions” (2007, 126). But if intuitions lack epistemic weight, what the folk think is of marginal philosophical significance.^6^ In fact, work in x-phi itself often catalyzes and confirms worries about the epistemic status of intuition that undermine its evidential role in philosophical theorizing.

To some degree, the probative power of empirical data and statistically rigorous analysis can help mitigate difficulties associated with the comparative reliability, track record, and irresolvable cross-population differences concerning intuitions [(i)–(iii)]. For example, x-phi can reveal when intuitions are largely based on biasing psychological mechanisms. But to the extent a positive program is committed to treating intuition, no matter how scrubbed of psychological bias and sources of cognitive error, as providing significant guidance for the second step, it remains vulnerable to the deficiencies of intuition-based philosophical methodology.

Despite the deficiencies of traditional approaches to evaluating conceptual content, concepts remain indispensable to acquiring evidence, evidentiary inference, and theory construction in philosophy and science. A defensible method for concept evaluation and determination is therefore still needed. Explication offers such a method. The next section clarifies x-phi’s important place within it.

## 3 Experimental Explication Preparation

Many theories and arguments that concern philosophers involve imprecise folk concepts used in everyday communication, and towards that end they are relatively unproblematic. But for the same reason these concepts constitute inadequate cognitive tools for scientific theorizing, they seem to serve theorizing in philosophy no better. As Kitcher aptly describes Carnap’s diagnosis of the challenge: “Carnap takes an important project of scientific philosophy to be the construction of systems of exact concepts that can better serve the purposes toward which older, vaguer, more confused forms of language have been directed” (2008, 113). As a general method for determining conceptual content, explication was developed to place the use of concepts in philosophy on as rigorous and secure a methodological footing as science.

Explication replaces or transforms a concept (the *explicandum*) typically drawn from “everyday language or…a previous stage in the development of scientific language” (Carnap 1950, 3) into another concept (the *explicatum*) guided by four desiderata: retain similarity of conceptual content with the explicandum, and increase precision, fruitfulness, and simplicity, the last being subordinate to the others. Scientific methodology supplies the benchmarks against which these desiderata and the concepts they employ should be understood. Similarity, for example, is construed quite weakly.^7^ Prioritizing precision and fruitfulness over strict preservation of conceptual content reflects methodology in science and as the unparalleled exemplar of epistemic success in human inquiry Carnap thought philosophy should follow suit. When concepts are characterized in science, antecedent conceptual content is readily sacrificed to achieve other ends, such as achieving empirical adequacy. Accepting the conventionality of simultaneity, abandoning species essentialism, and rejecting the frame-invariance of mass are well-known examples.

Precision for precision’s sake is not the agenda. Rather, enhancing precision usually enhances fruitfulness, which is the agenda. Among other things, precision is a reliable tool for ensuring the empirical adequacy of hypotheses, models, and theories: that the predictions they generate accord with observations. Without sufficiently precise concepts, it is difficult if not impossible to derive predictions from statements containing them. Without such predictions, in turn, statements cannot be confirmed or disconfirmed. Increasing precision also *usually* enhances mathematical rigor, measurability, testability, theoretical unification, etc.^8^ Achieving these objectives has a highly consistent, inductively well-supported track record of yielding the main currency in which science measures epistemic success: well-confirmed generalizations. For this reason Carnap accorded precision a central role in explication and measured fruitfulness in the same currency (1950, 7).

In sharp contrast with traditional conceptual analysis, explication sanctions significant deviation from a concept’s intuitive content to increase fruitfulness (Carnap 1950, 6–7). Retaining similarity is thus subservient to fruitfulness in explication, for at least three reasons. First, many (perhaps most) folk concepts and even some concepts found in science are problematically vague. Concepts such as ‘intelligence,’ ‘life,’ and ‘substance’ are so amorphous and imprecise it is unlikely any explication that retains a high degree of similarity would help discover well-confirmed generalizations. Second, uncritical talk of *the* concept X can also lull one into a false sense of conceptual unity, that there is in fact a unary X. Machery’s (2009) recent work indicates how problematic this can be. He argues individual concepts typically decompose into three distinct types of entity—exemplars, prototypes, and theories—that are used in different cognitive processes. The upshot is that the putatively singular concept X is more likely an amalgam of distinct bodies of information, employed in different ways.^9^ “The concept X” therefore embodies a dubious presupposition.

Third, many concepts possess content and encourage implications that would mislead rather than guide explication. For example, Griffiths et al. (2009) have shown ‘innateness’ conflates three dissociable features (fixity, typicality, and teleology). Explications of innateness constrained strongly by similarity would need to capture all three features and their interrelations. But since biological systems need not and often do not instantiate all three features, similarity-guided explications of innateness would likely be unfruitful. Recent studies in cognitive science reveal similar concerns about the concept ‘free will.’ They suggest free will is sometimes taken to require an “executive self”—a fictional entity operating above and beyond the mental states and capacities human agents possess (Knobe and Nichols 2010). Given these results, similarity-guided explications would hinder the burgeoning science of free will by precisifying something nonexistent. ‘Innate’ and ‘free will’ are far from isolated examples; many other concepts contain content explication should avoid. In this way, empirical results from x-phi help justify Carnap’s lax similarity criterion for explication.

These findings might make one wonder why intuitive conceptual content matters. Being tethered to imprecise explicanda appears to hinder, not advance, the development of fruitful explicata. But radical revisionism overlooks how folk concepts often describe features of the world and guide in theorizing about them, albeit rudimentarily. With its emphasis on precision and fruitfulness, explication endeavors to improve this functionality by developing more rigorous and systematic sets of concepts. To pinpoint the content that merits attempted preservation and the content that should be abandoned, however, a method for vetting explicanda is needed.

Carnap recognized this and argued:

[S]ince even in the best case we cannot reach full exactness, we must, in order to prevent the discussion of the problem from becoming entirely futile, do all we can to make at least practically clear what is meant as the explicandum…An indication of the meaning with the help of some examples for its intended use and other examples for uses not now intended can help the understanding. An informal explanation in general terms may be added. (1950, 4)

Unlike his detailed account of explication, in 1950 Carnap said very little about what methods would supply the needed clarification.^10^ Although the connection to explication was little discussed, the methodology he developed later in “Meaning and Synonymy in Natural Languages” precisely fits the bill. Guided by the view that “the assignment of an intension [i.e. meaning] is an empirical hypothesis which…can be tested by observations of language behavior” (1955, 37), Carnap described a method for uncovering intensions that involves presenting language users with a range of logically possible scenarios and asking them to make judgments regarding the concept in question. This obviously resembles the survey methodology of x-phi—indeed it warrants identifying Carnap as an early pioneer of x-phi—but Carnap remained open to methodological improvements and alternatives.^11^

With its insistence on using scientific methods to analyze empirical sources of information about concepts, x-phi complements Carnap’s (1955) data-driven methodology and embodies a key element in a defensible contemporary alternative to traditional conceptual analysis. X-phi has an especially valuable role to play in *explication preparation* (EP). Explicandum clarification, for example, is best achieved through empirically rigorous studies of the kind experimental philosophers conduct, which can:

(i) uncover regions of vagueness in extensions and intensions of concepts. For example, work on ‘intentional action’ reveals uncertainty about the amount of skill needed to perform an intentional action (Nadelhoffer 2005). And work on attributions of phenomenal states uncovers ambivalence about ascriptions of pain to systems that are part human and part robot (Heubner 2010).
(ii) reveal instances of conceptual pluralism underlying a notion. Mele and Cushman (2008) uncovered distinct patterns of disagreement across participants’ judgments about ‘intentional action,’ suggesting that more than one concept applies. Finding such pluralism is one key to diagnosing debates in philosophy that are “merely verbal” (Chalmers 2011).
(iii) discover sources of bias that influence intuitions. Weinberg et al. (2001) found that cultural differences influence judgments about ‘knowledge,’ and Schwitzgebel and Cushman (2012) found that presentation order influences judgments of professional philosophers and laypersons alike regarding several moral principles.
(iv) discover unpredictable (even if non-biasing) influences on conceptual judgments. Feltz and Cokely (2009) found that personality differences—specifically, introversion or extraversion traits—predict differences in judgments regarding ‘intentional action.’ Weigel (2011) found that differences in temporal distance—e.g. whether a particular judgment is imagined to occur *in a few years* or *a few days*—significantly influence intuitions about ‘free will.’ And Hitchcock and Knobe (2009) found that tacit application of salient norms impacts judgments about causation.
(v) outline a concept’s central features and its dependence relationships with other concepts. Work on ‘innateness’ reveals its central features and indicates the problematic relationships between them (Griffiths et al. 2009). And work on ‘free will’ has uncovered connections between ‘consciousness’ and capacities for agential behavior (Shepherd 2012).

Such significant insights over such a short period evince x-phi’s promise, and showcase the valuable contribution it can make to explication. Of course, the contribution x-phi makes will not determine, in any particular case, how explication should go. Explicative choices (e.g., choices about which features of concepts to preserve and which to abandon) will be guided in part by theoretical aims particular to the case at hand. Even so, x-phi’s contribution to such choices secures a positive philosophical payoff *independent* of contentious debates about intuition’s evidential status. The payoff is admittedly instrumental. Its value is not thereby made tenuous or trivial. At the basis of explication is an appeal to something (well-confirmed generalizations) whose epistemic credentials are unassailable, indispensable to science, and, crucially, independent of intuitive conceptual content. The more x-phi facilitates explication by helping clarify explicanda, the more x-phi participates in a compelling philosophical methodology. In this way EP supplies a cogent positive program for x-phi, one that connects x-phi to scientific practice (through explication) in a way naturalists should find salutary.^12^

A compelling new positive program for x-phi is an auspicious consequence of combining the two methodologies. There is, however, a pressing concern that reveals a potential limitation of our account. Recall Sosa’s claim that intuitions about normative truths of epistemology analogically function as observations do for science. One might contend that x-phi as explication preparation leaves this view unchallenged. That EP plays a legitimate role in the evaluation and determination of *empirical* concepts does not establish it plays a similar role for concepts with epistemically *normative* content. Empirical concepts serve descriptive theories that answer to a world amenable to empirical investigation. Methodologically, explication complements this agenda. But normative concepts serve normative theories and it is entirely unclear they answer to empirical evaluation beyond, perhaps, the familiar counsel that ought implies can. Perhaps the type of conceptual guidance explication provides simply does not extend to the normative domain.

This worry has bite. Of course, normativity comes in many stripes, and addressing them all exceeds the capacity of this paper. The next section develops the concern with respect to the specific normative domain explored by formal epistemologists. Section 5 defuses the worry, extends the relevance of x-phi assisted explication to concepts with normative content, and shows how explication can undergird methodology in formal epistemology.

## 4 Formal Epistemology and the Limits of Explication

Explication and formal epistemology emerged from a kindred dissatisfaction with standard philosophical methodology concerning epistemological issues. The standard approach emphasizes what intuitions and sometimes fantastical thought experiments seem to show about long-standing issues and the problematically vague concepts so often at their heart. Definite views are typically expected about whether, for instance, the possibility of being deceived in the matrix precludes knowledge possession or whether veridical perception in a barn-façade subdivision is justificatory. Beyond having such convictions, they are also taken to reliably reveal how knowledge, justification, and other normative epistemic concepts *should* be conceptualized. A convincing account of why intuition is trustworthy in this regard remains elusive and given its deficiencies in other areas—especially those in the purview of empirical study—there was ample appetite for alternative approaches to epistemic questions, which many formal epistemologists attempted to sate.

True to their common disposition, formal epistemology exemplifies explicative methodology. Precision is a priority for both, and enhancing precision is frequently prioritized over fidelity to intuition. As such, formal epistemologists generally eye the intuition-driven maneuvers prevalent in traditional epistemology with suspicion. In a book attempting to bridge the disciplines, Hendricks (2006, 16) captures the sentiment:

[W]hereas [mainstream epistemologists] often remain quite vague about the tacit assumptions and presuppositions of their conclusions, which are based on intuitions and folksy examples, [formal epistemologists] use intuitions and examples only to illustrate results obtained within a framework.

Axiomatic logics, probability calculi, and vetted methods of statistical inference are expected to ground epistemic claims and supplant intuitive judgments concerning the accessibility of possible worlds, thought experiments, and the like. The objective is putting epistemological analysis on the same rigorous and scrutable footing as other formal disciplines such as the mathematical and statistical sciences. With the clarity precision affords, Carnap hoped explication would put all of philosophy—including its empirical *and* normative domains—on such a secure footing. Formal epistemology is an attempt at one part.

Besides developing entirely new conceptual tools far removed from folk notions—different game-theoretic equilibria concepts, maximum entropy principles, etc.—formal epistemology also focuses on formulating precise surrogates for folk epistemic concepts, surrogates which often diverge markedly from entrenched meaning. For example, the logically demanding knowledge concept found in epistemic logics such as S4, and particularly S5, departs significantly from how human knowledge is usually conceptualized (see Stalnaker 2006). Meaning obviously changes if vague conceptual content is precisified, but further divergence is frequently sanctioned, just as explication counsels. A striking example is the enigmatic ingredient that transforms true belief into knowledge in non-Gettier cases according to traditional epistemology. It likely has no counterpart in Bayesian epistemology.^13^ The main obstacle to its integration is clearly vagueness, and paradoxes that emerge from attempts to render justification precise probabilistically suggest many epistemic concepts and issues will require reconceptualization in formal epistemology (see Shogenji 2012). Rather than inherit their imprecision, the hope is that problematically vague concepts can be replaced with a superior system of precise concepts. Formal epistemology endeavors to do for the theory of knowledge what game theory did for strategic thinking (see Binmore 2007).

Carnap’s own work speaks to the converging agendas of formal epistemology and explication. Carnap explicated several theoretical concepts—e.g. analyticity, entropy, semantic information—with the same high degree of precision and technical sophistication exhibited in formal epistemology. In fact, Carnap’s (1950) most detailed exposition of explication immediately precedes his explication of several core concepts of formal epistemology, including degree of confirmation and logical probability.

This confluence, the precision agenda of both, and their shared willingness to sacrifice intuitive conceptual content suggest explication constitutes the best methodology for formal epistemology and grounds its philosophical value. Carnap’s rationale for explication could then be cited to neutralize the criticism that formal epistemology, although technically intriguing, is largely philosophically irrelevant because it departs from the central concepts and concerns of traditional epistemology. Moreover, the rationale reflects scientific methodology and is compelling for philosophy of science (Justus 2012), so surely it confers the same support for formal epistemology. That Carnap placed no restriction on explication’s scope as a concept determination method and wielded it for epistemic concepts seems to clinch the case.

The issue is far from so straightforward. For Carnap, enhancing fruitfulness justifies the priority on increasing precision and licenses departures from intuitive conceptual content. But how should fruitfulness be understood? Carnap recognized two types. For empirical concepts, fruitfulness was cashed out in the incontrovertible currency of epistemic success: well-confirmed generalizations (see Sect. 3). For logical concepts, Carnap (1950, 7) simply stated that the currency is theorems without further elaboration. The more theorems an explication of a logical concept helps produce, the more fruitful it presumably is. Unfortunately, neither the empirical nor logical notions provide a defensible basis for explication in formal epistemology. The latter is especially problematic.

Start with the logical notion. Developing axiomatic logics to precisify and systematize epistemic concepts and their relationships is a common maneuver in formal epistemology (Hendricks 2006). Just as axioms are said to implicitly define mathematical concepts, axiomatic characterizations of epistemic concepts are intended to supply similarly rigorous explications. Beyond axioms, theorems in such systems also reveal information about concepts and their interrelations. Evaluating an explication therefore depends on the implicit definition axioms provide, what theorems hold, and what they say about a concept’s content. Proposed axioms in mathematics are evaluated similarly (see Easwaran 2008). But Carnap’s view that axiomatic explications should be judged by their facilitation of theorems provides very poor guidance. Simply counting the logical theorems facilitated is untenable: almost any axiomatic system would facilitate an infinite number. Moreover, explication is non-conservative in the terminology of formal theories of definition (Belnap 1993).^14^ It therefore may introduce inconsistencies into an axiomatic system. The vast number of “theorems” such inconsistencies would generate surely cannot redound to the fruitfulness of an explication. Not the number generated, but what theorems say seems crucial to evaluating an explication’s fruitfulness. Yet, if the pre-systematic, intuitive content of epistemic concepts is the proper benchmark for evaluating what the theorems say, the rationale for explication has largely been forgone. And since increasing precision encourages divergence from that content,^15^ it also seems to work against fruitfulness so construed.

As Sect. 3 explained, Carnap’s account of fruitfulness for empirical concepts is not similarly deficient. But the glaring problem for formal epistemology is that this account is inapplicable. Unlike empirical sciences, formal epistemology is not attempting to describe or represent features of the world in any straightforward sense. Rather, it is primarily a normative discipline.^16^ For example, Bayesian and Jeffrey updating specifies how beliefs *should* change when new evidence is acquired, not necessarily how they do. But relative to this goal there seems to be no counterpart to the well-confirmed generalizations that undergird explication as philosophical methodology. For formal epistemology, and normative disciplines generally, explication’s payoff is therefore unclear.

## 5 Fruitfulness for Formal Epistemology

The willingness to deviate from intuitive conceptual content to increase precision suggests a close kinship with explication, but some formal epistemologists see the kinship with traditional conceptual analysis. Hendricks (2006), for example, attempts to unify mainstream and formal epistemology under the banner ‘plethoric epistemology.’ For him, formal epistemology provides “systematic, rigorous and structured ways of conducting exactly what advocates of conceptual analysis claim epistemology is about” (2006, 162). In effect, formal epistemology is regimented conceptual analysis:

The value of local [i.e. formalized] analyses of epistemic concepts for the entire community of epistemologists lies in the regimentation of global intuitions about rationality, justification, reliability and cognitive strength; the fixation of their content and scope; and the creation of systematic manuals for their actual and limiting use (2006, 162).

Here the envisaged kinship is explicit; elsewhere it is tacit. Christensen notes that in practice, “Formal models of philosophically interesting concepts are tested largely by seeing how well they match intuitive judgments in particular cases” (1999, 460). Too much deviance from a concept’s intuitive content spells failure for formalizations that model it.

Recent formal treatments of the ‘coherence’ concept illustrate the point. Coherence is a relation between bits of evidence: the more coherent the evidence for a proposition, the more the evidence justifies believing it. Several formal coherence measures have been developed. Due in part to constraints drawn from conceptual analysis, none have garnered consensus. For example, Bovens and Hartmann seek to develop a coherence measure that respects intuitions about “our pre-theoretic notion of the coherence of an information set” (2003, 34). They do so by constructing a hypothetical scenario and three sets of information about that scenario labeled α, β and γ. Bovens and Hartmann claim that “Without having done any empirical research, we conjecture that most experimental subjects would indeed rank the information set in situation α to be more coherent than the information sets in either situations β or γ” (2003, 40). Akin to x-phi’s positive program, Bovens and Hartmann take intuitive accord with their conjecture to be substantive evidence for it. Siebel and Wolff argue similarly that failing to respect intuitions licenses “throw[ing] a significant number of probabilistic coherence measures overboard” (2008, 179). That successful formal measures of coherence should pass intuitive muster is unquestioned.

If preserving intuitive content is the goal, x-phi should be deployed. The empirically defensible methods of concept clarification x-phi offer apply straightforwardly to epistemic concepts. The information acquired can then guide attempted formalizations. And x-phi provides information not readily accessible from the armchair, by discovering influences and biases on conceptual judgments, instances of conceptual pluralism, etc. (see Sect. 3).

But for the reasons already discussed, it is not at all clear this should be the goal. The case for skepticism about intuition’s evidential role in philosophical theorizing is no less cogent in formal epistemology than outside it. Formal approaches to epistemological issues have several advantages, but formal precision alone furnishes no resources for addressing the evidential status of intuitions. Nor do intuitions seem to be the appropriate grist for *formal* epistemology. Scrupulously respecting intuitions about imprecise epistemic folk concepts seems misguided when pursuing the fruits of precisification.

Formal epistemology is better construed as an explicative enterprise and x-phi as explication preparation (EP) situates x-phi as an important component of this comprehensive methodology for prescriptive concept determination. But against this methodology, the problem discussed earlier arises (Sect. 4): an account of fruitfulness is required that licenses deviation from intuitive content for normative concepts. Carnap’s account of fruitfulness for empirical and logical concepts does not supply such an account.

In this connection, recognizing why Carnap chose well-confirmed generalizations as the measure of explicative fruitfulness for empirical concepts proves illuminating. Recall that explication is designed to reflect scientific methodology, and fruitfulness is intended to gauge epistemic success. There are various ways of evaluating scientific work, so why measure epistemic success by well-confirmed generalizations? Scientific consensus provides a compelling reason. Unlike other metrics—e.g. explanatory depth, mathematical rigor, parsimony—there is veritably no controversy that discovering well-confirmed generalizations constitutes genuine success. Most if not all empirical scientific work aims directly at uncovering such generalizations through experimentation and observation. And although theoretical scientific work sometimes concerns issues not immediately tied to discovering well-confirmed generalizations, they remain the goalpost by which theory is ultimately assessed.

Although consensus is much rarer among philosophers of science, on this specific issue there is surprisingly widespread agreement. There are disagreements about what some well-confirmed generalizations mean and what inferences they license: whether generalizations concerning so-called unobservables ground abductions about ontology is a prime example. But no party to these disagreements disagrees with scientists that well-confirmed generalizations are the principal currency for measuring epistemic success. Moreover, most other scientific virtues have well-confirmed generalizations at their base. Besides the close connection with empirical adequacy (see Sect. 3), Sober (1988) has shown that parsimony is only a defensible inference condition when it captures salient aspects of the phenomena being inferred about, which is in turn only evaluable if the relevant well-confirmed generalizations describing them are available. And various explanatory virtues (depth, force, scope) all depend on well-confirmed generalizations to supply the resources for *explanans* and targets for *explananda*. Well-confirmed generalizations are Carnap’s focus with fruitfulness to reflect these dependencies, and their privileged epistemic status in science.^17^

What is needed is a fruitfulness metric for the normative concepts of formal epistemology. Attempting to develop a *single* account of fruitfulness for this domain is probably foolhardy given the uncertainty and controversy about the nature of epistemic normativity. Fortunately, explication is a flexible tool. In spite of disagreement about the nature of epistemic normativity, explication can be fruitful for epistemic theorizing. We indicate how below.

### 5.1 Instrumental Rationality and Dutch Books

Consider Dutch Book Arguments (DBAs) concerning Kolmogorov’s axioms and Bayesian conditionalization. Whether synchronic or diachronic, these arguments reveal a dramatic breakdown of instrumental, means-ends reasoning. This is the source of their normative force.^18^ It’s not that bilked agents necessarily lose utility because they fail to grasp something intuitive about their degrees of belief or the bets offered, it’s that effective means-ends reasoning requires they adhere to certain norms. Explications that violated such norms—e.g. explicating ‘degree of belief’ or ‘coherence’ so that Kolmogorov’s axioms were violated—would be spectacularly unfruitful because they would utterly subvert effectively using those explicata to achieve one’s ends given one’s means. The empirical analog would be explicating ‘force’ such that mechanical laws no longer held, or ‘species’ such that evolutionary theory no longer accounted for their origin. Any epistemology, formal or otherwise, should guard against the kind of necessary utility loss DBAs procure.

This insight can be leveraged because the type of normative guidance DBAs provide has wider scope than is usually appreciated. Formal epistemology obviously concerns more than conforming to probability axioms and conditionalization conditions. Envisioning how formal epistemology would help with more formidable bookies clarifies the issue. Consider an infallible bookie with limitless powers of coercion to compel agents to take bets according to their credences. Relative to an agent’s credences, such a formidable bookie can compel bet-taking and would offer bets about states of the world that at best yield no loss to bet takers. Though hypothetical, minimizing loss against this bookie indicates how the deliverances of formal epistemology can be evaluated; with less adverse odds and coercive capability the world effectively functions as such a bookie relative to our ends. Achieving ends requires gathering and cultivating resources, identifying impediments, and developing means to avoid or overcome them. To aid this compulsory navigation, formal epistemology marshals tools from several sources: decision and game theory, Bayesian arguments, classical and non-classical logics, statistics, etc. Humans need and utilize these tools to different degrees, but we are all inescapably playing such a game against nature.

This perspective has philosophical precedent. It is a formal gloss on Goldman’s (1978) “epistemics.” But to our knowledge its application to formal epistemology is new, and it affords a notion of fruitfulness with attractive features:

(i) It delivers many seemingly nonnegotiable desiderata. Two main goals of epistemic agents are increasing their set of true beliefs and minimizing their set of false beliefs. Explications that advance these objectives supersede those that do not, all else being equal. And of course the bigger the set of true beliefs and the smaller the set of false beliefs, the better agents would fare against the bookie described above.
(ii) Since improving means-ends reasoning is the goal, intuitions (and our inherited conceptual scheme generally) have no privileged status. Rather, the emphasis is on better utilizing sources of beliefs and inference methods with reliable records of supporting instrumental reasoning. Unsurprisingly, science is the reliable source *sine qua non*.^19^ This ensures a thorough integration of formal epistemology and naturalized epistemology. For example, explications of epistemic concepts should consider how they might cohere with and ideally improve the statistical methods that deliver well-supported beliefs in the sciences. Similarly, whether an explication improves instrumental rationality depends on accurate identification of the psychological mechanisms that underpin human cognitive capabilities and how their various aberrations, biases, and shortcomings can be mitigated (see Bishop and Trout 2005). As naturalism counsels, what psychology reveals about human cognition is therefore a critical component of epistemology. Diagnosing these shortcomings and improving our native capabilities requires a clear account of what is possible were we more cognitively well-endowed. Formal epistemology endeavors to give such an account, thereby providing an ideal benchmark by which human cognition can be gauged and possibly enhanced.

It should be stressed that the story of what improves instrumental rationality is far from completed. Dutch book arguments for Kolmogorov axioms and conditionalization principles set a minimal standard. What further conditions share this status or help avoid less deleterious deficiencies—such as the epistemic frailty various reflection principles guard against (Briggs 2009)—is an active research area. One merit of this approach to epistemic normativity is that the results of this inquiry have a central rather than peripheral role within it.^20^

### 5.2 Distinctively Epistemic Fruitfulness

For some, instrumental rationality exhausts epistemic normativity (see Brössel et al. 2012 for discussion). But many maintain distinctively epistemic normativity cannot be reduced to instrumental rationality. Those formal epistemologists who seek to elucidate and apply distinctively epistemic norms—that is, norms about what ought (epistemically) to be believed, how beliefs ought (epistemically) to be updated, or how we ought (epistemically) to reason—might worry that explication is unhelpful given their objective.^21^ The worry is misguided. Deploying x-phi and explication in formal epistemology does not depend on any particular normative goal, framework, or theory. X-phi assisted explication is a tool, and prima facie the features that render it fruitful for scientific theorizing reviewed in Sect. 3 render it fruitful for (distinctively) epistemic theorizing. Explication does not compel specific epistemic norms and objectives, any more than it determines the fact that well-confirmed generalizations undergird progress in science. Instead, explication simply facilitates compliance with and achievement of these norms and objectives.

On the view that emerges, intuitions need not constitute the primary data for epistemic theories. Rather, epistemic norms might derive from broader theoretical considerations and goals, and empirical data about human psychology (of the sort x-phi offers) could indicate how a concept should be explicated. Admittedly, short of a comprehensive and compelling theory of epistemic normativity, no single account of fruitfulness against which to test proposed explications is available. But given its important but delimited function as a methodological tool, this poses no problem for explication. If anything, it is incumbent upon those committed to distinctively epistemic normativity to explain their notion of epistemic progress in much greater detail. Perhaps it would involve identifying merely verbal disagreements a la Chalmers (2011), discovering sources of bias or dependence relationships between epistemic concepts, and so on. If so, the argument in Sect. 3 applies and x-phi and explication offer valuable assistance. Formal epistemologists interested in elucidating distinctively epistemic normativity therefore need not worry that explication is somehow methodologically unfit for the task.^22^

## 6 Conclusion

Carnapian explication constitutes a naturalistic, data-driven, and epistemically compelling methodology for concept determination. We demonstrate the consilience between explication and a newer data-driven approach to the study of concepts, x-phi. X-phi has an important function within explication, a function we have called *explication preparation*. The upshot is two-fold. First, explication preparation constitutes a defensible positive program for x-phi. Second, x-phi supplies valuable data about the concepts targeted for explication.

As originally formulated, explication applied only to empirical and logical concepts. Given that normative concepts are the focus of much philosophical practice, one might worry that the methodology is of limited scope. We show that this worry is misguided. Explication can be fruitfully applied in at least one normative domain, formal epistemology. There is thus no general reason to think explication fails as a methodology for the determination of normative concepts.

## Footnotes

1 Partly responsible for this is the fact that philosophers remain divided over the nature of concepts. Some view concepts as abstract objects that serve as constituents of propositions (see Peacocke 1992); others view concepts as structured mental representations (see Margolis and Laurence 2007). We favor the latter, but the positions advanced below do not require endorsement of either view.

2 This interpretation of the use of intuitions in conceptual analysis has been denied by Cappelen (2012). In interesting discussions of Cappelen’s position, Bengson (2014) and Chalmers (2014) make compelling cases that philosophers do in fact frequently rely on intuitions as evidence. This is not to deny that other sources of evidence (e.g., arguments) are also important.

3 We remain neutral on the exact nature of intuitions: whether intuitions are beliefs, dispositions to believe, intellectual seemings with a characteristic phenomenology, or whatever (see Pust 2012). The main issue here is the evidential status of intuitions.

4 Nadelhoffer and Nahmias (2007, 126) describe a similar split. The minor differences between the characterizations are left unaddressed.

5 It is this justificatory role that we find particularly problematic. We recognize that intuitions sometimes function as useful heuristics for theory development. On this point, see Bengson’s (2014) interesting discussion of the way intuitions sometimes serve what he calls “prompting” and “problematizing” functions.

6 It may be relevant for other purposes. For example, important policy implications might follow from results about folk beliefs, especially about value-laden concepts. And philosophers should certainly care about policy implications. The critical target here is the relation of folk intuitions to theories about the nature of empirical phenomena (e.g. free will).

7 Retaining similarity was a matter of degree for Carnap; minimum thresholds were not stipulated; and he only required that “most of what previously was said with the [explicandum] can now be said with the help of the [explicatum]” (1950, 6). In fact, “considerable differences” (1950, 7) in content were permitted. Explication may even change extensions. The similarity condition is therefore quite weak, but few further details were provided. For more on Carnap’s similarity requirement and the epistemic rationale underlying explication see Justus (2012).

8 Carnap recognized that precisification is sometimes unfruitful, citing some psychological research at the time as an example (1950, 14). For a detailed exposition of the role of precision in empirical sciences see Hempel (1969). With a biological example, Justus (2012) examines how precision can be enhanced in different ways in explication.

9 Machery suggests this plurality warrants elimination of the notion ‘concept,’ but the data he discusses do not necessitate eliminitivism. This plurality might simply stem from dissociable cognitive systems that are nevertheless legitimate sources of conceptual competence and judgment (Piccinini 2011).

10 Carnap very briefly discussed a few examples but they are rather uninformative, especially about what general clarificatory methods are appropriate. For example, Carnap suggested the explicandum ‘salt’ could be clarified by noting that one intends the concept as used “in the household language” (1950, 5).

11 Carnap (1950, 8) approvingly cited Arne Naess’ (1953) monograph, then in preparation, which developed different methods for measuring ambiguity, precision, vagueness, and for testing the synonymity of expressions involving judgments over paired sentences coupled with statistical analysis.

12 Explication also allows negative program proponents to side-step a potential problem. Following Weinberg’s (2007) analysis of the worry, Ichikawa (2012, 744) argues a viable negative program must explain how the critique of armchair methodology “does not generalize to their preferred practices, including, at least, a great deal of science, as well as the use they themselves put to philosophical considerations in their own arguments against armchair philosophy.” But the concern dissolves once x-phi is understood as EP. A robust critique of intuition plainly fails to threaten explication because in it intuitions play no substantive justificatory role.

13 Two prominent and comprehensive introductions to Bayesian epistemology say almost nothing about justification (Howson and Urbach 1993; Bovens and Hartmann 2003).

14 In axiomatic contexts, a definition conservatively extends a theory T in a language L to $\overset{´}{T}$ in an extended language Ĺ if for every sentence ϕ of L, T proves ϕ iff $\overset{´}{T}$ proves ϕ. Put informally, conservative definitions do not facilitate proofs of anything new (other than derivative consequences of extending the language). Acquiring new knowledge in this way is precisely the point of explication.

15 No divergence need occur if the contours of epistemic concepts are extraordinarily sharp, such that increasing precision (through axiomatization or otherwise) more accurately reflects their true structure. On this view the rationale for increasing precision in epistemology would mirror methodology in physics and most other sciences: precision simply facilitates more accurate description, in this case of conceptual content rather than physical properties and processes. But this is highly implausible. How competent natural language speakers actually understand and employ terms representing epistemic concepts—and what cognitive psychology generally reveals about the fluid nature of concepts (Machery 2009)—shows this ambitiously precise account of our epistemic conceptual apparatus is mistaken. Of course, these results are not conclusive if epistemic concepts are abstract objects that serve as constituents of propositions (Peacocke 1992), *and* the realm of such conceptual abstracts is precisely structured. We believe this view of concepts (and conceptual structure) is untenable, but cannot address this complex issue here.

16 Formal epistemology can also address descriptive issues about human cognition (e.g. Schupbach 2011). Analyses with this focus share goals similar to psychology and Carnap’s rationale for explication of empirical concepts applies.

17 Carnap (1950, 7) simply gauged fruitfulness in terms of the production of well-confirmed generalizations, but his explications (and the scientific methodology they reflect) demonstrate that well-confirmed generalizations contribute differentially, not equally, to fruitfulness. Explications that facilitate numerous well-confirmed, but nevertheless trivial generalizations fare poorly on fruitfulness. Generalizations with wider scope contribute more, as do generalizations that better catalyze discovery of further generalizations. Beyond these plausible general principles, Carnap’s reticence about fruitfulness likely reflects a pragmatic perspective: fruitfulness depends on the particular purposes for which explication is employed. Carnap’s naturalism and scientific orientation tempers this pragmatism (see Richardson 2008), but a pragmatic perspective provides a clear way to generalize explication to normative concepts (see below).

18 Some have argued diachronic DBAs are flawed (e.g. Christensen 1991; Arntzenius 2003). Briggs (2009) convincingly responds to these criticisms.

19 Science is the *sine qua non* we know. Other unimagined conceptual systems may be as or more efficacious at achieving ends. This is demonstrated particularly clearly in debates about the new-found merits of alternative, nontraditional logics, including dialetheic logics (see Priest et al. 2004).

20 Epistemic normativity concerns a narrower subset of the plethora of ends humans generally value. It is an open question whether the drive to enhance precision and willingness to abandon intuitive conceptual content advances those ends. For example, Stich (1990) defends a much broader pragmatic view of how human cognitive systems should be evaluated. Explications fruitful for formal epistemology may be infertile on this expansive understanding of epistemology’s scope. Blissful ignorance and intellectual myopathy may make one happier and even more biologically fit than the tortured polymath, but the icy logic of the coercive epistemic bookie punishes the former while rewarding the latter.

21 For example, Joyce (1998) constructed a non-pragmatic version of the DBA that established the following: credences that violate Kolmogorov’s axioms are invariably less accurate representations of the state of the world than they could be if they conformed, for a reasonable measure of representational accuracy.

22 Given persistent disagreement regarding the nature of moral normativity, as well as the goals of ethics and best ways of achieving them, the same points plausibly apply to explication of moral concepts. Explication is a flexible tool, and we should expect many of its positive features noted above to transfer to the moral domain. Unfortunately, discussing how specific accounts of fruitfulness might work for ethical explication is a substantive project that cannot be pursued here.
